# Attention Modulates Visual-Tactile Interaction in Spatial Pattern Matching

**DOI:** 10.1371/journal.pone.0106896

**Published:** 2014-09-09

**Authors:** Florian Göschl, Andreas K. Engel, Uwe Friese

**Affiliations:** Department of Neurophysiology and Pathophysiology, University Medical Center Hamburg-Eppendorf, Hamburg, Germany; University of Chicago, United States of America

## Abstract

Factors influencing crossmodal interactions are manifold and operate in a stimulus-driven, bottom-up fashion, as well as via top-down control. Here, we evaluate the interplay of stimulus congruence and attention in a visual-tactile task. To this end, we used a matching paradigm requiring the identification of spatial patterns that were concurrently presented visually on a computer screen and haptically to the fingertips by means of a Braille stimulator. Stimulation in our paradigm was always bimodal with only the allocation of attention being manipulated between conditions. In separate blocks of the experiment, participants were instructed to (a) focus on a single modality to detect a specific target pattern, (b) pay attention to both modalities to detect a specific target pattern, or (c) to explicitly evaluate if the patterns in both modalities were congruent or not. For visual as well as tactile targets, congruent stimulus pairs led to quicker and more accurate detection compared to incongruent stimulation. This congruence facilitation effect was more prominent under divided attention. Incongruent stimulation led to behavioral decrements under divided attention as compared to selectively attending a single sensory channel. Additionally, when participants were asked to evaluate congruence explicitly, congruent stimulation was associated with better performance than incongruent stimulation. Our results extend previous findings from audiovisual studies, showing that stimulus congruence also resulted in behavioral improvements in visuotactile pattern matching. The interplay of stimulus processing and attentional control seems to be organized in a highly flexible fashion, with the integration of signals depending on both bottom-up and top-down factors, rather than occurring in an ‘all-or-nothing’ manner.

## Introduction

Our natural environment is inherently multisensory and requires continuous simultaneous processing and accurate combination of inputs from the different sensory systems to create meaningful percepts. Two important factors influencing crossmodal integration are stimulus congruence and attention. While crossmodal stimulus congruence is thought to facilitate cognitive processing in a bottom-up manner, top-down attention allows us to dynamically select from the available information and process relevant aspects while ignoring irrelevant others. For example, imagine yourself in a low light situation trying to pick the right key from your key chain. If the perceived visual and tactile features of the different keys at your hand are sparse, it might be the combination of the two modalities that enables you to identify the correct key. Moreover, actively paying attention to a single modality as opposed to congruent or conflicting crossmodal information is likely to influence the outcome of your search. Though clearly relevant for organizing and streamlining the flow of perceptual information, few studies have focused on the influence of modality specific selective attention versus distributed crossmodal attention for multisensory processing (e.g. [1]–[4]). In the present study, we focus on interactions between vision and touch and the question how performance is affected by crossmodal stimulus congruence under different attentional demands.

Extensive research in the field of multisensory perception has established a set of principles governing the interaction between different sensory modalities. Temporal and spatial proximity between stimuli occurring in multiple modalities have been shown to maximize multisensory integration, measured in terms of firing patterns of single multimodal midbrain neurons in anesthetized animals [5], [6]. These observations at the single cell level led to the formulation of the ‘temporal’ and ‘spatial rule’ of multisensory integration. The relevance of these principles for human multisensory perception has been investigated extensively in behavioral and neuroscientific studies in the last decades. Presenting stimuli at approximately the same time or location often enhances multisensory integration leading to improvements in detection, discrimination or localization performance and faster response latencies [7]–[18]. For a detailed overview of behavioral evidence for the ‘temporal’ and ‘spatial rule’ of multisensory integration see also [19].

Beside spatiotemporal concordance, other factors influencing multisensory interaction have been identified in recent years, among those the so-called crossmodal correspondences (for a review see [20]) and contextual congruence [2], [3], [21]–[27]. For example, in a study using a redundant cue feature discrimination task, it was demonstrated that semantically congruent audiovisual stimulation led to improved behavioral performance as compared to either unisensory case [24]. Incongruent pairs of stimuli in turn were associated with performance decrements relative to both unimodal discrimination tasks. In one of the few studies on attentional modulations of multisensory integration, Mozolic et al. [3] extended these findings. They showed that if participants directed attention to both modalities, crossmodal congruence facilitated responses. In contrast, selective attention to either the visual or the auditory modality apparently prevented the integration of semantically matching stimuli and resulted in a lack of performance gains for congruent multisensory stimuli. However, the authors could not report any (originally hypothesized) effects of selective attention on cross-modal distraction caused by incongruent pairs of stimuli. Similar behavioral results were reported in an ERP study by Talsma et al. [4]. The authors observed enhanced performance for audiovisual stimulation as compared to auditory and visual stimulation alone only if participants distributed attention to both channels. On the other hand, this study found evidence from ERPs that multisensory integration took place even when attention was directed to a single modality, though this process appeared to be delayed. Overall, the specific circumstances under which attention enhances or impairs congruence-related performance gains remain unclear.

In an attempt to shed further light on the interplay of bottom-up stimulus congruence and top-down attentional demands, we utilized a crossmodal matching paradigm requiring the identification of concurrently presented visual and tactile spatial patterns [28]. The stimuli consisted of patterns composed of three dots, which were presented briefly on a visual display and simultaneously to the tip of the index finger by means of a Braille stimulator. Unlike most related studies, stimulation in our paradigm was always bimodal with only the allocation of attention being manipulated between conditions. In separate blocks of the experiment, participants were instructed to either (1) focus on a single modality to detect a specific target pattern, (2) pay attention to both modalities to detect a specific target pattern, or (3) to explicitly evaluate if the patterns in both modalities were congruent or not. This last condition was included to represent the situation in which crossmodal congruence is actively searched for—as in the “key chain example” above. The design realized here is novel in concurrently manipulating stimulus congruence and attention in a balanced manner to further elucidate the relation of top-down and bottom-up factors in multisensory processing. To our best knowledge, most of the preceding studies considering the tactile modality in a multisensory context have focused on temporal order judgments, detection or localization tasks. Our paradigm in contrast requires the identification and the cross-modal comparison of spatial patterns and thereby adds substantial new facets to multisensory research.

Our hypothesis was that crossmodal stimulus congruence (i.e. matching patterns) would influence behavioral performance differentially depending on the focus of attention. In line with previous work on audiovisual integration [2], [3], we expected congruence driven gains in behavioral performance also for our visual-tactile matching paradigm. We assumed those differences to be more pronounced under divided attention demands as compared to modality specific focused attention. For incongruent pairs of stimuli, however, distributing attention across sensory modalities could enhance crossmodal distraction and hence degrade performance. In the crossmodal matching condition, which explicitly required attention to operate on a combination of features from both modalities, we also expected stimulus congruence driven advantages to be reflected in improved behavioral performance.

## Methods

### Participants

A total of 49 healthy volunteers were monetarily compensated for participating in the study. Ten participants could not take part in the actual experiment due to poor performance in a training procedure that was conducted to familiarize participants with the tactile stimuli (39 remaining, 24 female, mean age 24.4, range 19–31). All participants had normal or corrected to normal vision, were right-handed and reported no history of neurological or psychiatric disorders.

### Ethics statement

The Ethics Committee of the Medical Association Hamburg approved the current study. In accordance with the Declaration of Helsinki written informed consent was obtained from every participant prior to the experiment.

### Setup and stimuli

Participants were seated in a light attenuated chamber in front of a 21-inch CRT computer monitor (distance to screen 110 cm), their right hand comfortably placed on a custom-made board containing the tactile stimulator. The board was located in front of the participants on their right side.

In the experimental session, stimuli were always delivered concurrently in the tactile and the visual modality. Tactile stimuli were presented to the right index fingertip via a Braille stimulator (QuaeroSys Medical Devices, Schotten, Germany). The Braille stimulation cell had a matrix of four rows by two columns with eight independently controllable pins, each 1 mm in diameter with a spacing of 2.5 mm.

**Figure 1:**
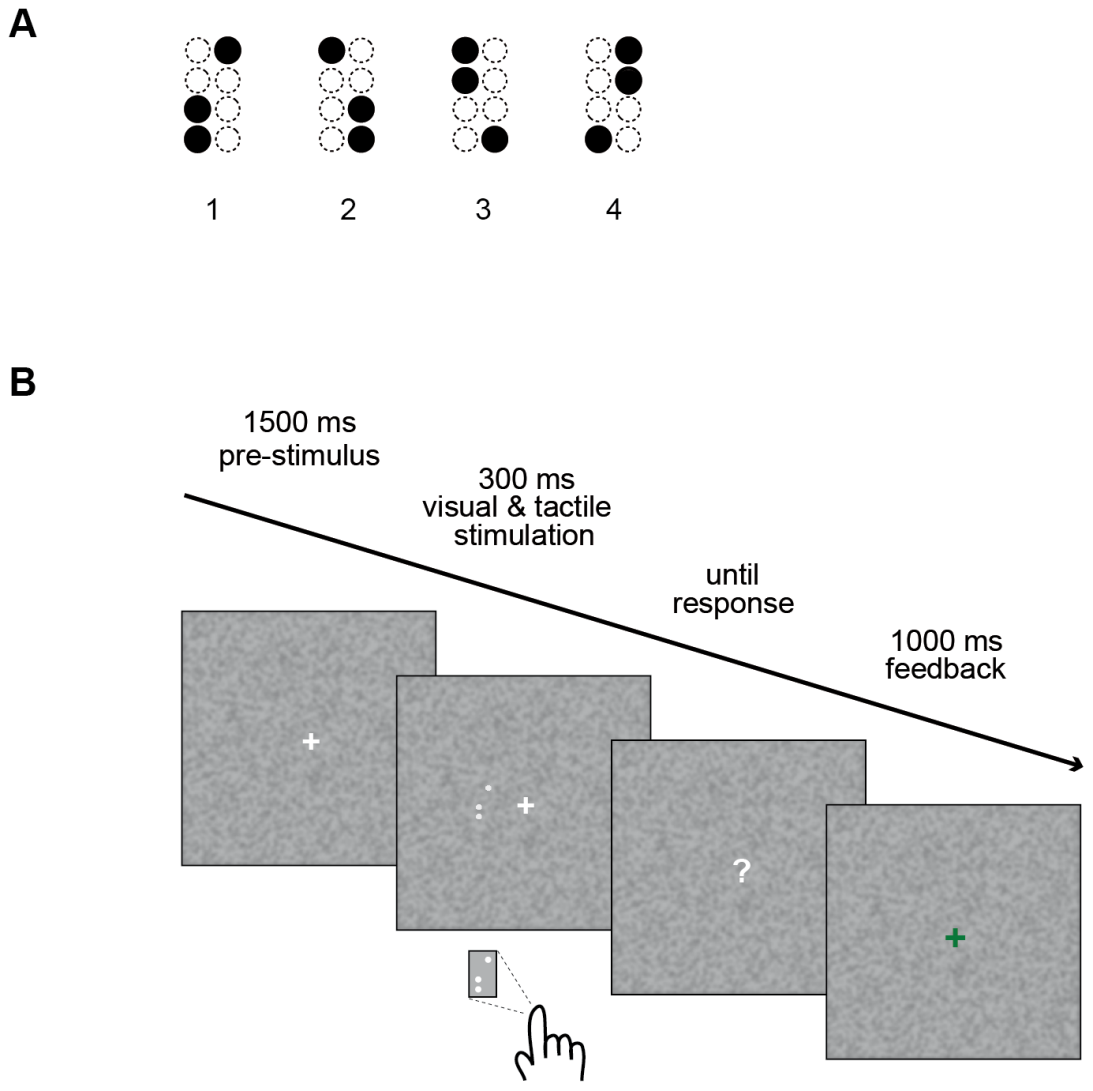
Schematic representation of the visual-tactile detection task. **A**: The four pattern stimuli used in our experiment. **B**: The trial sequence. After a pre-stimulus interval of 1500 ms, visual and tactile stimuli were presented concurrently for 300 ms, followed by a question mark indicating that responses could be given. After button press, every trial ended with visual feedback (1000 ms).

The stimulus set consisted of four spatial patterns formed by three dots each (Figure 1A). For tactile stimulation the three pins were raised concurrently with an amplitude of approximately 1.5 mm, held elevated for 300 ms, and then lowered again. The spatial configuration of the patterns as well as the stimulation parameters were chosen based on a pilot study to ensure good performance in pattern recognition at relatively short presentation times (approximately 80% correct answers in a tactile delayed-match-to-sample task).

Visual stimuli were designed analogously to the tactile patterns with respect to configuration, spatial relations of the dots and timing. Light grey dots were presented on a noisy background (Figure 1B). The complete visual pattern subtended 3.5°×2.5° of visual angle and was presented 2.5° left of a central fixation cross. Visual presentation was lateralized to ensure comparability to a planned neurophysiological study.

### Training paradigm

Before the actual experiment participants were trained in the tactile domain by means of a delayed-match-to-sample task. Each training trial started with a delay interval of 2000 ms, followed by the presentation of a sample stimulus (for 300 ms) that was chosen pseudo-randomly from the stimulus set. After another delay interval of 1000 ms and the presentation of a second tactile stimulus (again for 300 ms) participants were asked to judge whether the two consecutively presented patterns were the same or not. Participants responded via button presses with the left index or middle finger on a response box (Cedrus, RB-420 Model, San Pedro, USA) as soon as a question mark appeared on the screen (after another 1000 ms after stimulus offset). At the end of each trial, participants were given visual feedback (a green ‘+’ or a red ‘–’) informing them about the correctness of their response (1000 ms). Throughout the training session, participants wore earplugs to prevent the hearing of sounds generated by pin movement in the Braille cells.

Eight pairs of tactile stimuli (two patterns always being presented sequentially within a single training trial) were designed such that each of the four patterns appeared four times with an equal number of congruent stimulus pairs (i.e., containing two identical patterns) and incongruent stimulus pairs (i.e., containing two different patterns). One training block consisted of 16 trials and a minimum of five blocks had to be completed. Training performance was considered to be sufficient if matching accuracies reached at least 80% within two consecutive blocks (starting with block number four). Ten out of 49 participants did not meet this criterion within 15 blocks and were not included in the actual experiment.

### Experimental paradigm

The goal of the current study was to investigate the effect of crossmodal stimulus congruence under different attentional demands. To this end, we confronted participants with four different attentional tasks while they were presented with concurrent visual and tactile patterns: (1) *focused visual attention*, (2) *focused tactile attention*, (3) *divided visuotactile attention*, and (4) *visuotactile matching*. These conditions were defined as follows.

*Focused visual attention and focused tactile attention*: In the focused attention conditions the task was to detect a target pattern in one modality only (either the visual or the tactile one). Target patterns were defined at the beginning of the block by repeatedly presenting (four times) one of the four patterns either on the computer monitor (*focused visual attention*) or on participants' right index finger (*focused tactile attention*). In the following experimental trials participants had to decide for the pattern presented in the attended channel whether it matched the target stimulus or not and press one of two response buttons accordingly.

*Divided visuotactile attention*: In the third condition, participants were instructed to detect targets in both modalities. As for the focused attention conditions, one of the four patterns was introduced at the block start as the target stimulus. However, in this condition patterns were presented four times in both channels simultaneously. The task now was to deploy attention to both modalities and detect visual or tactile targets. Participants were notified that targets could appear in the visual or the tactile channel alone, in both or in neither of the two. Again one button had to be pressed for target stimuli, another one for non-targets (the assignment of keys was the same as in conditions one and two).

*Visuotactile matching*: The fourth condition also required distributing attention to vision and touch. Participants were asked explicitly to compare patterns across modalities and had to judge whether the two patterns were the same or not. Half of the participants were instructed to press the ‘target’-button whenever they detected a match between the two patterns (and the ‘non-target’-button for non-matches). For the other half incongruent pairs were defined as targets, implying that the ‘target’-button had to be pressed for the non-matching patterns (and the ‘non-target’-button for matches). As for the other conditions, targets were introduced initially by sequentially presenting either the four congruent (‘match as target’ group) or the four incongruent pairs of stimuli (‘non-match as target’ group).

Responses were given via button press with the left index or middle finger. Speed and accuracy of the answers were emphasized likewise for all conditions and visual feedback was given in every trial.

The temporal structure of the task was the same for the different conditions and is depicted in Figure 1B. Each trial began with participants fixating on a white cross, displayed centrally on a noisy background for an interval of 1500 ms. Following fixation, visual and tactile patterns were presented with synchronous on- and offset for 300 ms. Subsequently, a white question mark appeared on the screen signalling that responses could be given. After button press, a green ‘+’ sign or a red ‘–’ sign, respectively, informed participants about the correctness of their decisions (1000 ms). All in all one trial lasted approximately 3500 ms.

The experiment comprised 512 trials in total. Half of all stimulus pairs presented were congruent and each of the four patterns appeared with same frequency in the visual and tactile modality (128 times). Each of the four conditions consisted of 128 trials, half of them containing target patterns (64) and – at the same time – half of them containing congruent pairs of stimuli. For the two focused attention conditions, targets were presented equally often in congruent and incongruent pairs (32 each). In the *divided visuotactile attention* condition, half of the target trials (32) contained congruent pairs (i.e. targets in the visual and the tactile channel), a quarter (16) visual targets and another quarter (16) tactile targets only. The *visuotactile matching* condition was composed equally by congruent and incongruent pairs of stimuli (and thereby targets and non-targets).

Target definition for every pattern was counterbalanced as well (each pattern appearing four times as tactile, four times as visual and four times as combined visual/tactile target). Stimulus presentation was organized within blocks, each block only containing eight trials and always repeating the same block sequence throughout the experiment: *focused visual attention*, *focused tactile attention*, *divided visuotactile attention* and *visuotactile matching*. Randomization was realized for every pattern and condition (e.g. all the stimulus pairs for pattern 1 as a visual target were randomized) and sequences for a single block were drawn afterwards. Short breaks intermitted the experiment every 16 blocks (i.e. every 128 trials). Key mapping (for ‘target’ and ‘non-target’-buttons) was counterbalanced across participants. As in the training session, participants wore earplugs to mute sounds associated with the tactile stimuli. For both the training and the experimental session, Presentation software (Neurobehavioral Systems, version 16.3) was used to control stimulus presentation and to record participants' response times (RT) and accuracies.

### Data analysis

All data collected in our study are available in Dataset S1. Participants' averaged accuracies and mean response times (RT) were calculated for the four conditions after removing RT outliers (300 ms as a lower limit and mean RT +2 standard deviations as an upper limit). Additionally, we computed inverse efficiency scores (IES) for each participant, providing a measure for overall performance in which RT are inflated in proportion to error rates [18], [29], [30]. IES are interpreted in the same way as correct RT and are especially useful if error rates vary across experimental conditions [31]. For our target detection paradigm, mean RT is divided by the averaged accuracies (ACC):
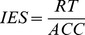
(1)


To further illustrate the modulation of multisensory interaction between visual and tactile modality by attention and stimulus congruence, we adopted a measure termed ‘multisensory response enhancement’, which is a descriptive measure relating performance in unimodal conditions to performance in crossmodal ones [18], [32]. Here we use a modification of this index to depict facilitation effects in multisensory interaction resulting from stimulus congruence and define multisensory congruence enhancement (MCE) as follows:
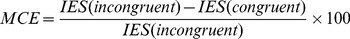
(2)


The MCE score relates overall performance (using IES) for incongruent target cases (target only present in one channel) to performance for congruent target cases (targets present in both channels) and is calculated for the different attention manipulations. Increased performance for congruent pairs (i.e. smaller IES) results in larger positive MCE values. Low MCE values indicate small differences between the performance on incongruent and congruent pairs of stimuli. MCE was also computed for the *visuotactile matching* condition to illustrate differences in detection performance for congruent and incongruent pairs of stimuli. Additionally, detection performance was represented by sensitivity estimate d' [33]. We calculated d'-estimates in each condition from hit and false alarm rates for target and non-target stimuli, separately for congruent and incongruent cases. Hits were defined as correct identifications of target patterns (two measures were calculated for targets appearing in either congruent or incongruent pairs of stimuli); responses to non-targets on the other hand were categorized as false alarms (again measured for congruent and incongruent pairs). If not stated otherwise, all reported MCE scores and d'-estimates were significantly different from zero (one-sample t test).

Data for conditions *focused visual attention*, *focused tactile attention* and *divided visuotactile attention* were analysed using 2 (congruence) × 2 (attention) repeated measures analyses of variances (ANOVAs) to determine whether d'-estimates as well as IES differed depending on congruence (congruent versus incongruent) or attention (focused versus divided). Separate ANOVAs were calculated for the comparison of visual and tactile targets appearing in congruent and incongruent stimulus combinations under different attention manipulations.

Furthermore, we computed differences between d'-scores for congruent and incongruent pairs of stimuli for both attention manipulations. To illustrate possible congruence facilitation effects graphically, we followed a procedure recommended by Loftus and Masson [34] using 95% confidence intervals. Confidence bounds, which do not include zero, represent reliable differences – corresponding to one-sample t tests results.

To analyse detection performance in the *visuotactile matching* condition, IES were subjected to a 2 (congruence) × 2 (target definition) repeated measures ANOVA with congruence (congruent versus incongruent) as within-subjects factor and target definition (match as target versus non-match as target) as between-subjects factor. Moreover, d'- estimates were calculated for both groups (the ‘match as target’ group as well as the ‘non-match as target’ group) separately and compared using an independent-samples t test.

## Results

A total of 39 participants completed the training procedure successfully within 6.75 blocks (108 trials) and 13.6 minutes on average. Overall, 79.8% of all experimental trials were answered correctly. Employing a criterion of 300 ms as a lower limit and mean RT +2 standard deviations as an upper limit for the detection of RT outliers, 4.9% of the correct trials were discarded. Average accuracies, mean response times (RT) and IES, MCE scores and d'-estimates for the visual-tactile detection task are displayed in Table 1. For reasons of clarity and economy concerning the presentation of our results, we focus on IES, MCE scores and d'-estimates in the following. (For a comparison of RT and IES analyses see the last paragraph of the Results section).

**Table 1:** Mean accuracies and response times (ms), averaged inverse efficiency scores (IES), multisensory congruence enhancement (MCE) scores and d'-estimates with standard errors (SE) for the visual-tactile detection task (see Methods section for details).

ACC (SE)			RT (SE)		IES (SE)		MCE	d' (SE)	
congruent		incongruent	congruent	incongruent	congruent	incongruent		congruent	incongruent
**Visual**									
Focused	0.91 (0.01)	0.89 (0.01)	722 (33)	723 (31)	800 (39)	814 (40)	1	2.8 (0.08)	2.58 (0.11)
Divided	0.92 (0.01)	0.86 (0.02)	912 (39)	1031 (48)	993 (44)	1230 (69)	16	1.98 (0.09)	1.58 (0.11)
**Tactile**									
Focused	0.84 (0.01)	0.74 (0.02)	871 (34)	956(42)	1043 (44)	1308 (57)	19	1.64 (0.07)	0.98 (0.07)
Divided	0.92 (0.01)	0.6 (0.03)	912 (39)	1364 (70)	993 (44)	2492 (177)	55	1.98 (0.09)	0.62 (0.11)
**Matching**									
Match as target	0.77 (0.02)	0.57 (0.03)	1406 (85)	1516 (100)	1861 (132)	3496 (574)	27	0.98 (0.15)	
Non-match as target	0.71 (0.02)	0.64 (0.03)	1552 (59)	1591 (71)	2304 (143)	2864 (278)	8	0.99 (0.16)	

IES displayed here are means of ratios (IES  =  RT/ACC) calculated within single subjects.

### Visual targets

**Figure 2:**
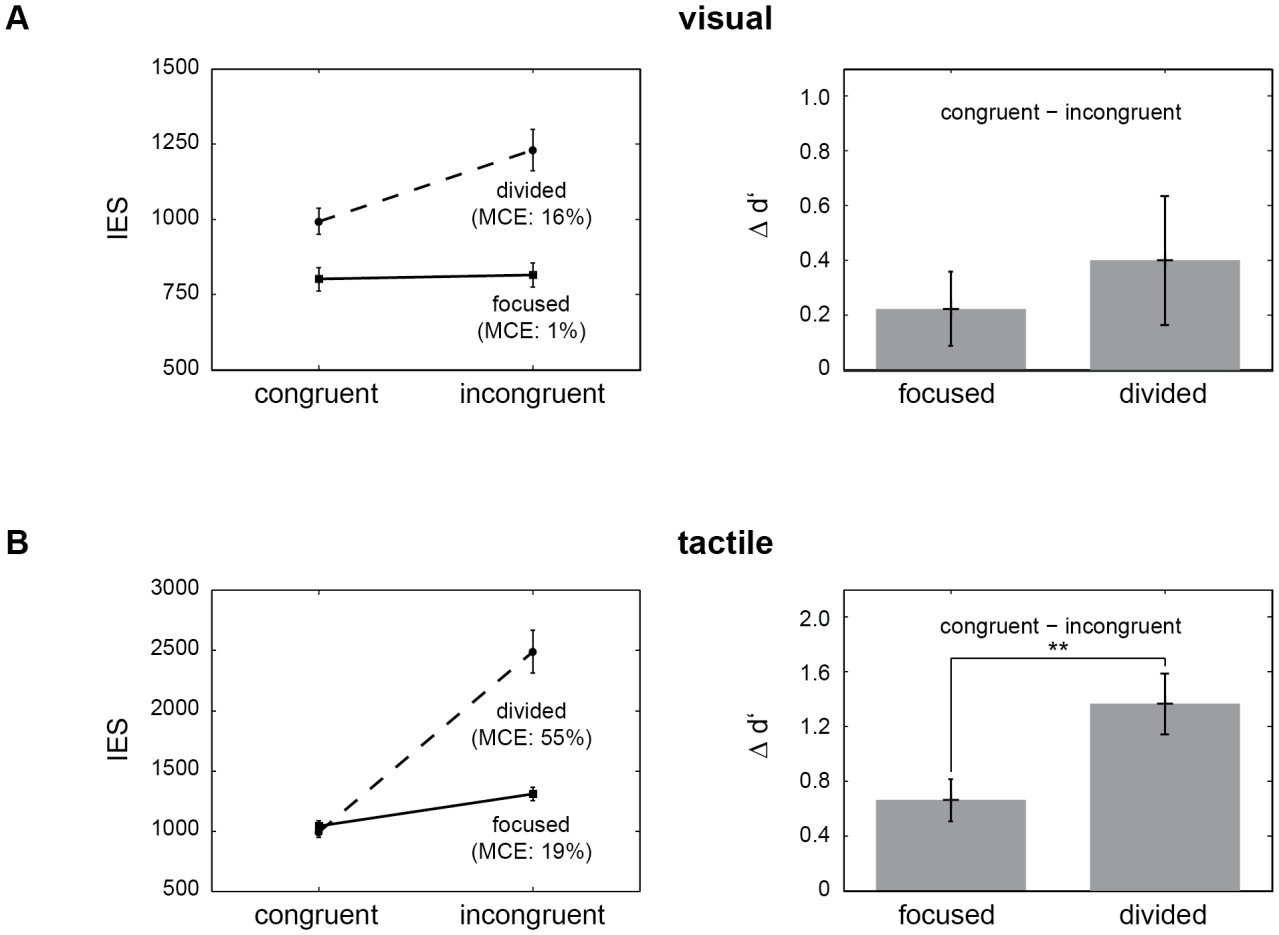
Influence of pattern congruence and attention on detection performance of visual (A) and tactile targets (B). **A**: (Left) Mean inverse efficiency scores (IES), shown with standard errors (SE) for the detection of visual targets in congruent and incongruent pairs of stimuli. The solid line mirrors performance for the focused attention manipulation, the dashed line for divided attention. Multisensory congruence enhancement (MCE) scores are displayed in brackets. Smaller IES for congruent pairs of stimuli illustrate the congruence facilitation effect in detection performance. This advantage is more prominent when attention is distributed across vision and touch, which is further illustrated by MCE scores. (Right) Difference plots for the d'-estimates comparing values for congruent and incongruent target cases under focused (left) and divided attention (right). The higher difference of d'-estimates for divided attention implies that stimulus congruence driven advantages are more pronounced when attention is distributed across the visual and the tactile modality. Nonetheless, stimulus congruence also results in improved behavioral performance for the focused attention manipulation. Error bars indicate 95% confidence intervals and confidence bounds not including zero represent reliable differences. **B**: Inverse efficiency scores (IES) and d'-differences for tactile targets. Again, multisensory congruence enhancement (MCE) scores are displayed in brackets and error bars in the right chart correspond to 95% confidence intervals. IES and d'-differences illustrate congruence driven benefits in behavioral performance. Of note, the congruence facilitation effect, as expressed in MCE scores and d'-differences, is significantly bigger for divided attention.

See Figure 2A for a graphical presentation of the main results concerning visual targets. Collectively, 89.8% of the visual targets were detected successfully. The ANOVA of IES for the detection of visual targets using the factors *congruence* (congruent versus incongruent) and *attention* (focused versus divided) revealed significant main effects of *congruence* (*F*_1, 38_ = 19.95, *p*<0.01) and *attention* (*F*_1, 38_ = 116.54, *p*<0.01). Critically, we also found a significant *congruence* x *attention* interaction effect (*F*_1, 38_ = 25.1, *p*<0.01), such that differences between congruent and incongruent target cases were relatively bigger under divided as compared to focused attention (*t*_38_ = 5.01, *p*<0.01, paired sample t test). Post hoc analysis of the main effects showed that IES for congruent pairs of stimuli (as compared to incongruent pairs) were only smaller under divided attention demands (*t*_38_ = 4.94, *p*<0.01; no significant difference for focused attention, *t*_38_ = 0.84, *p* = 0.4) whereas detection performance was higher in the focused attention manipulation (as compared to divided attention), for congruent (*t*_38_ = 8.78, *p*<0.01) as well as incongruent pairs of stimuli (*t*_38_ = 9.07, *p*<0.01). The analogous ANOVA for d'-estimates yielded comparable results with significant main effects for *congruence* (*F*_1, 38_ = 18.49, *p*<0.01) and *attention* (*F*_1, 38_ = 107.12, *p*<0.01) but no significant interaction (*F*_1, 38_ = 2.07, *p* = 0.16). Post hoc analysis of d'-scores revealed a stimulus congruence effect for both attention manipulations, focused (*t*_38_ = 3.3, *p*<0.01) as well as divided (*t*_38_ = 3.43, *p*<0.01). Thus, congruent visual-tactile stimuli led to better overall detection performance (measured with IES and d'- estimates) compared to incongruent stimulation. This congruence facilitation effect was more prominent under divided attention, which is also illustrated by the MCE scores. Across participants, congruent stimulation in the detection of visual targets produced a decrease of 1% in IES for focused attention (which was not significantly different from zero; *t*_38_ = 0.51, *p* = 0.62) as compared to 16% for divided attention. The contrast between MCE scores for focused versus divided attention was significant (*t*_38_ = 5.24, *p*<0.01, paired sample t test).

### Tactile targets

See Figure 2B for an illustration of the main results concerning tactile targets. Across participants, 76.1% of all tactile targets were detected. Analysis of IES for the detection of tactile targets revealed significant main effects of *congruence* (*F*_1, 38_ = 107.7, *p*<0.01) and *attention* (*F*_1, 38_ = 39.2, *p*<0.01). The interaction effect for *congruence* x *attention* was significant as well (*F*_1, 38_ = 51.82, *p*<0.01). As for the visual target detection, this effect was driven by differences between congruent and incongruent target cases being more pronounced under divided attention (as compared to focused attention, *t*_38_ = 7.2, *p*<0.01, paired sample t test). For post hoc analysis of main effects, paired t tests were computed as well and showed that stimulus congruence (as compared to incongruent presentation) enhanced detection performance significantly for focused (*t*_38_ = 8.3, *p*<0.01) and divided attention (*t*_38_ = 8.94, *p*<0.01). For the analysis of attention related influences on response behavior, paired t tests yielded a significant result for the comparison of IES for incongruent pairs of stimuli under focused and divided attention (*t*_38_ = 6.84, *p*<0.01), which was not the case for the congruent pairs (*t*_38_ = 1.49, *p* = 0.15). Results from the analysis of d'-estimates are consistent as far as the significant main effect of *congruence* (*F*_1, 38_ = 197.54, *p*<0.01) and the interaction of *congruence* x *attention* (*F*_1, 38_ = 31.99, *p*<0.01) are concerned. Post hoc comparison showed that differences between congruent and incongruent pairs were more distinct under divided attention (t_38_ = 5.66, p<0.01, paired sample t test). However, there was no significant main effect of *attention* (*F*_1, 38_ = 0.02, *p* = 0.88). Summing up, congruent stimulus presentation enhanced detection performance also for tactile targets. Again, we do find a strengthening of this stimulus congruence facilitation under divided attention demands. A benefit of stimulus congruence in the detection of tactile targets is also reflected in MCE scores. Congruent as compared to incongruent stimulation decreased IES by 19% in the focused attention manipulation and by 55% even under divided attention. Comparing MCE scores between the two attention manipulations yielded a significant result (*t*_38_ = 10.89, *p*<0.01, paired sample t test).

### Matching

**Figure 3:**
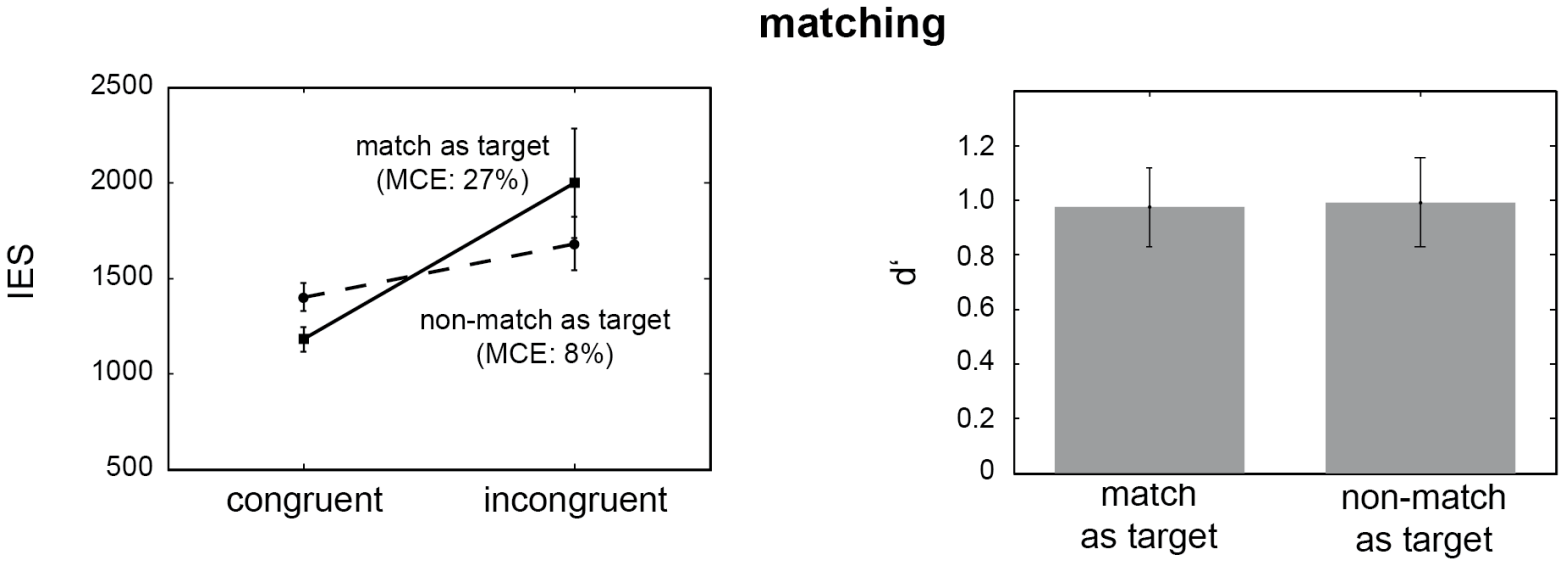
Influence of pattern congruence and target definition in the visuotactile matching condition. (Left) Mean inverse efficiency scores (IES), shown with standard errors (SE) for the detection of congruent and incongruent pairs of visual-tactile stimuli. The first group of participants (solid line) treated matching stimulus pairs as targets, whereas for the second group (dashed line) incongruent, non-matching pairs were defined as targets. Multisensory congruence enhancement (MCE) scores are displayed in brackets. IES indicate that stimulus congruence enhances detection performance, especially if participants treat congruent pairs as targets (‘match as target’ group). The MCE scores further support this finding. (Right) Mean d'-scores (with SE) for the ‘match as target’ (left) and the ‘non-match as target’ group (right). The d'-estimates do not differ between the two groups indicating comparable detection performance.

Results of the matching condition are depicted in Figure 3. The amount of accurately detected stimulus pairs was 67% for the ‘match as target’ group, and 67.5% for the ‘non-match as target’ group, respectively. Analysis of IES for the *visuotactile matching* condition yielded a significant main effect for the within-subjects factor *congruence* only (*F*_1, 37_ = 8.09, *p*<0.01; neither the between-subjects factor *target definition* nor the interaction effect were significant: *F*_1, 37_ = 0.03, *p* = 0.86 and *F*_1, 37_ = 1.94, *p* = 0.17, respectively). Post hoc paired t tests showed that the stimulus congruence driven advantage in detection performance was only significant for the ‘match as target’ group (*t*_19_ = 2.52, *p* = 0.02; no significant difference was obtained for the ‘non-match as target’ group, *t*_18_ = 1.4, *p* = 0.18). Averaged d'-estimates did not differ between the ‘match as target’ and the ‘non-match as target’ group (*t*_37_ = 0.08, *p* = 0.94, independent-samples t test). MCE scores for the *visuotactile matching* condition further support the notion that stimulus congruence enhances detection performance, especially if participants treat congruent pairs as targets (‘match as target’ group). IES were decreased by 27% in this case. Though there was an average reduction in IES for the ‘non-match as target’ group of 8%, the according values did not significantly differ from zero (*t*_18_ = 0.97, *p* = 0.35). The comparison of MCE scores for the two groups (‘match as target’ versus ‘non-match as target’) trended to significance (*t*_37_ = 1.74, *p* = 0.09, independent-samples t test).

### Comparison of RT and IES analyses

To make sure that IES accurately represent performance, we also analysed RT and accuracy data separately and obtained largely comparable result patterns. According to Townsend and Ashby [30], the IE metric's only assumption is a linear relationship between correct RT and error rates. Here, the correlation between correct RT and errors (1 – ACC) was *r*_37_ = 0.71 (*t_37_* = 2.63, *p* = 0.02), supporting the linearity assumption [30]. Results for the analysis of RT and IES were essentially equivalent. Only the main effect of the within-subjects factor congruence in the matching task trended to significance for RT (*F_1, 37_* = 3.61, *p* = 0.07), whereas it was significant for IES (*F_1, 37_* = 8.09, *p*<0.01). Significance patterns obtained for IES reflected those of RT and accuracies in most points. Observed discrepancies were the following: For visual targets, the main effect of attention was only mirrored in RT (*F_1, 38_* = 153.67, *p*<0.01), not in accuracies (*F_1, 38_* = 1.43, *p* = 0.24). Similarly, for the detection of tactile targets the effect of attention only trended to significance (*F_1, 38_* = 3.41, *p* = 0.07) for accuracies, whereas it was significant for RT (*F_1, 38_* = 50.06, *p*<0.01).

## Discussion

In the current study, we investigated the influence of modality specific selective attention versus divided attention on multisensory processing in a visual-tactile setting. To this end, we used a pattern matching paradigm requiring the identification of simultaneously presented visual and tactile spatial configurations under different attentional demands. We found that crossmodal stimulus congruence resulted in improved performance in all attention conditions but – as expected – those advantages were more pronounced for distributed attention. For visual and tactile targets likewise, detection was faster and more accurate when pairs of stimuli were congruent. These congruence driven advantages were enhanced when attention was distributed across sensory modalities as compared to selectively attending a single channel. Finally, even when participants were required to evaluate congruence explicitly, the detection of matching respectively non-matching pairs of visual and tactile patterns was facilitated by stimulus congruence.

Our results are in line with several findings from studies on the integration of contextual features involving vision and audition. Using spoken and written nouns in a target detection task, Mishra and Gazzaley [2] could show that semantically congruent audiovisual stimuli (as compared to visual stimuli alone) led to quicker and more accurate detection performance and that this congruence facilitation was more pronounced for distributed audiovisual attention as compared to focused visual attention (only for reaction times). Similarly, Mozolic et al. [3] found reaction times in a visual-auditory discrimination task to be shortened for congruent pairs of stimuli in relation to either unisensory case and modality specific selective attention to attenuate performance gains produced by semantically matching stimuli. Complementing these audiovisual studies with compatible evidence from the visual-tactile domain, we find congruence driven improvements in behavioral performance that are further enhanced if attention is divided across sensory channels. Noteworthy in this context are the different analyses of these effects. Whereas Mishra and Gazzaley [2], as well as Mozolic et al. [3] computed differences between performance on bimodal and unimodal stimuli to illustrate integration effects, we derived pattern congruence driven advantages by contrasting congruent and incongruent pairs of stimuli. One advantage of the ‘classical approach’ to compare multimodal to unimodal stimulation is the possibility to directly quantify multisensory enhancement. On the other hand, stimuli in our natural environment are rarely presented in isolation [35]. In the current study, stimulation always happened in a bimodal manner with only the attentional focus shifting.

For incongruent pairs of stimuli, Mishra and Gazzaley [2] reported interference effects under focused visual attention (with accuracies for incongruent audiovisual pairs being diminished compared to visual targets) that were resolved with dividing attention across modalities. Both, the described effects of interference resolution as well as the congruence enhancement under divided attention were accompanied by reduced neural processing of auditory and visual components. The authors explained this finding with enhanced efficacy of sensory neural processing during distributed relative to focused attention. Mozolic et al. [3], in contrast, reported no comparable effects for incongruent stimulus combinations on a behavioral level. In our study, however, we encountered performance on incongruent pattern combinations to be diminished under divided attention (as compared to modality specific focused attention), for visual and tactile targets. In other words, we found stronger effects of crossmodal distraction when dividing attentional resources, as hypothesized by Mozolic et al. [3]. One important factor that probably contributes to these diverging results is differences in task demands. Attention is particularly likely to influence multisensory [36], [37] as well as unisensory processing under high perceptual demands (for a classic example in the visual domain see [38]). Whereas the studies reviewed above used highly salient audiovisual material, the visual and tactile pattern stimuli in our experiment were perceptually more ambiguous. This notion is also supported by the larger congruence-related performance gains we observed for tactile targets as compared to visual targets. In other words, tactile targets were comparatively more difficult to detect and profited most from congruent visual stimulation under focused as well as under distributed attention instructions. Alternatively, the congruence-related improvements for the detection of tactile targets could also be interpreted in the light of different sensory processing delays for vision and touch. To disentangle these two interpretations of ‘task difficulty’ and ‘task timing’, systematically varying stimulus onset asynchronies for visual and tactile patterns might be a promising approach for future studies. To increase comparability of the detection of visual and tactile targets in our paradigm, attempts were made to align task difficulties. To this end, difficulty of the visual task was increased by embedding the stimuli in noisy backgrounds, increasing the eccentricity and reducing the size of the patterns. In addition, the tactile stimuli were optimized to facilitate detection. It should be noted that our data provide clear evidence that even though the detection of visual targets was easier, congruent tactile stimulation was still effective in improving detection performance.

Analysis of the behavioral data of the current study mostly showed parallel trends for inverse efficiency scores (IES) and d'-estimates, i.e. higher target detection rates were usually accompanied by lower reaction times. Some effects, however, were driven by RT rather than accuracy on the task, namely the interaction of *congruence* and *attention* for the detection of visual targets and the main effect of *attention* for tactile targets. For the *visuotactile matching* condition, target definition did not result in differences between the two groups (‘match as target’ versus ‘non-match as target’). The main effect of *congruence* (with lower IES for the detection of congruent pairs of stimuli) arose predominantly from differences in the ‘match as target’ group, whereas in the ‘non-match as target’ group congruent stimulation was associated with a non-significant reduction of IES by 8%. Of note, assuming target definition to explain differences found in behavioral performance, one would have expected a reversed pattern of results, with improved detection rates for incongruent pairs of stimuli as compared to congruent ones.

A descriptive examination of our two divided attention conditions, namely concurrent detection of targets in two sensory channels versus explicit matching of patterns in two modalities, yielded different temporal signatures. The larger response latencies in the matching condition most probably reflect additional processing required for explicitly evaluating the relation between the crossmodal stimuli. Therefore, it is tempting to speculate that these behavioral signatures might reflect different neural processes of interaction between top-down attentional factors and bottom-up stimulus processing. To clarify this issue, alongside related questions concerning the interplay of attention and multisensory integration at distinct early and late processing stages, electrophysiological recordings could be a fruitful approach for further research [4], [37].

Overall, our findings show that stimulus congruence is generally advantageous in crossmodal pattern matching. Such congruence-related enhancement effects are larger when attention is distributed across vision and touch rather than if attention is directed to a single modality. For incongruent pairs of stimuli, behavioral performance is improved for modality specific selective attention as compared to divided attention, possibly because of a reduction in crossmodal distraction. Taken together, our results suggest that the interplay of stimulus processing and attentional control is organized in a highly flexible fashion, with the integration of signals depending on both bottom-up and top-down factors, rather than occurring in an ‘all-or-nothing’ manner.

## Supporting Information

Dataset S1 **Raw data of the visual-tactile detection task.** Dataset S1 contains the following information: **column 1**…subject ID; **column 2**…trial number; **column 3**…task (1: focused visual attention, 2: focused tactile attention, 3: divided visuotactile attention, 4: visuotactile matching); **column 4**…target pattern (for matching task (4) always zero, as targets were defined as congruent or incongruent pairs of stimuli); **column 5**…tactile pattern; **column 6**…visual pattern; **column 7**…congruent (1: congruent, 0: incongruent); **column 8**…target present (in attended channel(s); 1: yes, 0: no); For the matching task (4), either congruence was defined as target (subjects with ID 1 to 26), or incongruence was defined as target (subjects with ID 27 to 49); **column 9**…subject's response (1: target detected, 0: no target detected); **column 10**…accuracy (1: correct, 0: incorrect); **column 11**…response time (ms); **column 12**…target for matching condition (0: match is target, 1: non-match is target). See Methods section for details.
(TXT)
