# Correction: Attention Modulates Visual-Tactile Interaction in Spatial Pattern Matching

**DOI:** 10.1371/journal.pone.0115930

**Published:** 2014-12-12

**Authors:** The PLOS ONE Staff

There are errors in Table 1. Please see the correct Table 1 here.

**Table 1:** Mean accuracies and response times (ms), averaged inverse efficiency scores (IES), multisensory congruence enhancement (MCE) scores and d'-estimates with standard errors (SE) for the visual-tactile detection task (see Methods section for details).

	ACC (SE)		RT (SE)		IES (SE)		MCE	d’ (SE)		
	congruent	incongruent	congruent	incongruent	congruent	incongruent		congruent		incongruent
**Visual**										
Focused	0.91 (0.01)	0.89 (0.01)	722 (33)	723 (31)	800 (39)	814 (40)	1	2.8 (0.08)		2.58 (0.11)
Divided	0.92 (0.01)	0.86 (0.02)	912 (39)	1031 (48)	993 (44)	1230 (69)	16	1.98 (0.09)		1.58 (0.11)
**Tactile**										
Focused	0.84 (0.01)	0.74 (0.02)	871 (34)	956(42)	1043 (44)	1308 (57)	19	1.64 (0.07)		0.98 (0.07)
Divided	0.92 (0.01)	0.6 (0.03)	912 (39)	1364 (70)	993 (44)	2492 (177)	55	1.98 (0.09)		0.62 (0.11)
**Matching**										
Match as target	0.77 (0.02)	0.57 (0.03)	1406 (85)	1516 (100)	1861 (132)	3496 (574)	27		0.98 (0.15)	
Non-match as target	0.71 (0.02)	0.64 (0.03)	1552 (59)	1591 (71)	2304 (143)	2864 (278)	8		0.99 (0.16)	
